# Effects of Different Astaxanthin Sources on Fillet Coloration and Energy Deposition in Rainbow Trout (*Oncorhynchus mykiss*)

**DOI:** 10.1155/2024/1664203

**Published:** 2024-03-25

**Authors:** Xiaoxue Meng, Fumei Yang, Lulu Zhu, Lingli Zhan, Toru Numasawa, Junming Deng

**Affiliations:** ^1^College of Fisheries, Guangdong Ocean University, Zhanjiang 524088, China; ^2^Kunming Biogenic Co. Ltd., Kunming 650220, China

## Abstract

A 9-week feeding trial was conducted to investigate the effects of different dietary sources of astaxanthin on fillet coloration, texture, and nutrient composition in rainbow trout (*Oncorhynchus mykiss*). Eight diets were formulated to contain 0, 25, 50, 75, 100, and 125 mg/kg astaxanthin from wall-broken *Haematococcus pluvialis* (WBHPA), 100 mg/kg astaxanthin from wall-unbroken *H. pluvialis* (WUHPA), or chemically synthesized astaxanthin (CSA). Each diet was fed to triplicate groups of rainbow trout (mean initial weight of 561 g) twice daily (07:00 and 17:00) to apparent satiation for 9 weeks. Results showed that at the 100 mg/kg astaxanthin inclusion level, the CAS group had higher fillet gross energy, dorsal fillet redness, and dorsal fillet color card score compared to the WBHPA-100 group, with both being higher than the WUHPA group (*P* < 0.05). Fillet astaxanthin content, dorsal fillet yellowness, and lateral line redness and yellowness did not differ significantly between the CSA and WBHPA-100 groups (*P* > 0.05), but were higher than the WUHPA group. When WBHPA was used, the inclusion of 50–100 mg/kg decreased fillet lightness but increased fillet redness, while better fillet texture was served at 75–125 mg/kg. Dietary 25–125 mg/kg WBHPA inclusion increased fillet astaxanthin and gross energy concentrations, with minor effects on fatty acid compositions of fillet. Inclusion of over 100 mg/kg astaxanthin regardless of source decreased fillet threonine and serine contents, and the WBHPA-100 group had lower fillet glycine and alanine contents compared to the control group (*P* < 0.05). In conclusion, CSA had the most significant impact on fillet coloration and energy deposition in rainbow trout, while WUHPA had the least favorable effect. Additionally, the wall-breaking treatment of *H. pluvialis* can improve the effect of astaxanthin on fillet coloration and nutrient composition in rainbow trout, with a recommended dose range of 75–100 mg/kg.

## 1. Introduction

Rainbow trout (*Oncorhynchus mykiss*) is a significant carnivorous cold-water fish, with production reaching 959,600 tons in 2020 [1]. The reddish or pink color of salmonids fillet is a crucial quality evaluation criterion that can greatly appeal to consumers. The color and texture of fillet in certain fish species and crustaceans are closely linked to their carotenoid composition (especially astaxanthin) and content [2–5]. Since fish lack the ability to synthesize astaxanthin *de novo*, they rely on external sources to maintain their natural pigmentation [6, 7]. In intensive farming settings, fish face considerable pressure and heavily rely on compound feed. It is essential to incorporate additives with prebiotic effects, such as astaxanthin, to ensure a balance between nutritional utilization, growth performance, and health status of farmed fish.

Astaxanthin can be derived naturally or through chemical synthesis. Natural astaxanthin is typically found in an esterified form, while chemically synthesized astaxanthin is in a less stable free form [8]. Additionally, natural astaxanthin is predominantly in a full cis structure, whereas synthetic astaxanthin is generally in a trans structure. Synthetic astaxanthin comprises three optical isomers, with approximately 25% being levorotatory (3S, 3S′), 25% dextrorotatory (3R, 3R′), and 50% racemic (3R, 3′S or 3S, 3′R). On the other hand, astaxanthin derived from *Haematococcus pluvialis* possesses a 100% levorotatory structure (3R, 3R′), resulting in a more potent antioxidant capacity. Moreover, the ratio of astaxanthin esters extracted from *H. pluvialis* (70% monoester, 25% diester, and 5% monomer) closely resembles that found in aquatic animals, potentially facilitating the utilization of astaxanthin extracted from *H. pluvialis* in aquatic animal feed [9–11].

*H. pluvialis*, a freshwater green microalga, is known for its commercial value due to its ability to naturally accumulate high concentrations of astaxanthin [9, 12]. However, before organisms can benefit from this astaxanthin, it must first undergo hydrolysis to break down the esterified form present in *H. pluvialis* during the digestion process [13]. Additionally, the digestibility of *H. pluvialis* is hindered by its microalgae cell wall, particularly in fish [14], leading to reduced availability of natural astaxanthin. A previous study comparing fillet astaxanthin concentrations in rainbow trout fed *H. pluvialis* versus synthetic astaxanthin [13] found lower levels in those fed *H. pluvialis*, possibly due to the presence of the algae cell wall. Therefore, there is a need to develop strategies to enhance the bioavailability of algal astaxanthin.

The objective of this study was to investigate the effects of different dietary sources (wall-broken *H. pluvialis*, wall-unbroken *H. pluvialis*, and synthetic astaxanthin) and doses of astaxanthin on fillet coloration, texture, and nutrient composition in rainbow trout. The findings of this research could provide valuable insights for improving astaxanthin management and optimizing its use in fish feed formulations.

## 2. Materials and Methods

### 2.1. Astaxanthin Sources

The sources of astaxanthin in this study included wall-broken *H. pluvialis* (WBHPA, purity 1.5%, provided by Kunming Biogenic Co., Ltd., Kunming, China), wall-unbroken *H. pluvialis* (WUHPA, purity 4.5%, provided by Kunming Biogenic Co., Ltd., Kunming, China) and chemically synthesized astaxanthin (CSA, purity 10%, provided by DSM (China) Limited). The WBHPA was derived from WUHPA through a process of high-pressure homogenization involving a cooling water system and high-pressure homogenizer.

### 2.2. Experimental Diets

The basal diet utilized in this study consisted of 40% crude protein and 18% crude lipid (Table S1). Eight experimental diets were formulated to contain 0, 25, 50, 75, 100, and 125 mg/kg astaxanthin from WBHPA, 100 mg/kg astaxanthin from WUHPA, and CSA. These were respectively labeled as control, WBHPA-25, WBHPA-50, WBHPA-75, WBHPA-100, WBHPA-125, WUHPA, and CSA. All ingredients were finely ground to a powder using a 60 *μ*m mesh, then mixed with fish oil, soybean oil, and soybean lecithin, followed by the addition of approximately 30% distilled water. The mixture was then processed into 3-mm diameter pellets using a pellet feed maker (KS-180, Jiangsu Jinggu Rice Mill Co., Ltd., Jiangsu, China). Subsequently, the pellets were dried at a constant temperature of 40°C for 12 hr and stored at −20°C until use.

### 2.3. Experimental Fish, Feeding Procedure, and Sampling

The initial body weight of the rainbow trout was 561.49 ± 2.17 g. To acclimate to the experimental conditions, all fish were housed in the experimental setup for 1 week and fed a commercial diet. Prior to the feeding trial, 720 fish were fasted for 24 hr and then randomly distributed into 24 cement tanks, with each group having triplicate tanks. Each tank (2 × 2 × 1.5 m^3^) was stocked with 30 fish and fed twice daily (07:00 and 17:00) to apparent satiation for 9 weeks. The water, after aeration and disinfection, circulated through a system with mechanical and biological filtration media and a UV lamp at a rate of 10 L/min. Throughout the experiment, the water temperature was maintained between 14−18°C with continuous aeration and natural photoperiod. Dissolved oxygen levels ranged from 7.8–9.2 mg/L, pH levels from 6.7–7.7, total ammonia nitrogen from 0.04–0.07 mg/L, and nitrite from 0.02–0.04 mg/L.

Following a 24-hr fasting period at the end of the feeding trial, three fish were randomly collected from each tank, anesthetized using eugenol (1 : 12,000; Shanghai Reagent Corporation, Shanghai, China), and euthanized by spinal cord severance. The fresh dorsal fillets (2.0 × 2.0 × 2.0 cm^3^) above the lateral line on both sides were immediately for fillet texture analysis. Simultaneously, the remaining dorsal fillet was collected and placed in liquid nitrogen, and stored at −76°C for subsequent analyses of proximate composition, fatty acid and amino acid profiles. The skinless fillet between the lateral line and dorsal fin of rainbow trout was taken for skin color measurement after blotting excess surface water with absorbent paper.

The experimental procedures were approved by the Institutional Animal Care and Use Committee of Guangdong Ocean University, and all protocols were strictly followed in accordance with their guidelines.

### 2.4. Chemical Analysis

The diets and fillet proximate composition analysis were conducted in accordance with the standard methods outlined by the Association of Official Analytical Chemists [15]. Briefly, the moisture content was determined by subjecting samples to oven-drying at 105°C until constant weight, the protein content was assessed by measuring the nitrogen (N × 6.25) content using the Kjeldahl method, the lipid content was carried out using the Soxhlet method, while the ash content was determined through incineration at 550°C. Gross energy was determined using the oxygen bomb calorimeter (ZDHW-6, Hebi Huatai Electronics Co., Ltd., Henan, China). Fatty acid composition of fillet was analyzed using gas chromatograph following the procedure described by Meng et al. [16]. Briefly, the fatty acids in freeze-dried samples were reacted with KOH–methanol and HCL–methanol, extracted with hexane, and then analyzed. The concentration of amino acids in fillet was determined using an amino acid analyzer (Hitachi L8900; Hitachi, Tokyo, Japan) as described by Deng et al. [17], after hydrolysis with 6 N HCl at 110°C oven for 24 hr. Astaxanthin level in the diet, serum, and fillet were quantified using high-performance liquid chromatography in accordance with the national standard of China (GB/T 31520-2015).

### 2.5. Analysis of Fillet Color

The redness (*a* ^*∗*^), yellowness (*b* ^*∗*^), and lightness (*L* ^*∗*^) of fillet were measured using a Roche colorimetric card and a WSC-S colorimeter on the side of the fillet facing the spine. Measurements was taken on both sides of each fish.

### 2.6. Analysis of Fillet Texture

The texture of fresh fillet was assessed immediately after collection using a TA-XTPlus Texture Analyzer equipped with a P/36R cylindrical detector. The analysis included a 5-s measurement pause, a compression rate of 60%, a trigger force of 5 g, and a constant speed of 2 mm/s. The instrument calculated six texture parameters (hardness, springiness, cohesiveness, adhesiveness, gumminess, resilience, and chewiness). Additionally, tenderness was measured using a tenderness meter, and the pH value was determined with a pH meter.

For the water-holding capacity assessment, a 5-mm-thick fillet (W_1_) was weighed and compressed between layers of filter paper under 35 kg of pressure for 5 min. After removing the filter paper, the fillet sample was reweighed (W_2_) to calculated the water-holding capacity using the formula:

(1) $$\begin{array}{c} \text{Water-holding capacity}\left(\%\right)=\left(\frac{1-\left({\text{W}}_{1}-{\text{W}}_{2}\right)}{{\text{W}}_{1}}\right)\times 100\%. \end{array}$$

### 2.7. Statistical Analysis

The normality and homogeneity of variance were assessed before conducting one-way analysis of variance (ANOVA) in SPSS 17.0 for Windows. Percentage data were firstly arcsine transformed for analysis. Tukey's multiple range test was used to identify significant differences among groups, with differences considered significant when *P* < 0.05. The data are presented as mean ± standard error.

## 3. Results

### 3.1. Fillet Proximate Composition

Within the dietary WBHPA supplementation groups, the fillet ash content was significantly (*P* < 0.05) lower in the WBHPA-75, WBHPA-100, and WBHPA-125 groups compared to the control group, with the WBHPA-75 group showing the lowest value (Table 1). Additionally, dietary WBHPA supplementation led to a significant (*P* < 0.05) increase in fillet gross energy content, reaching its peak in the WBHPA-75 group.

At the 100 mg/kg astaxanthin inclusion level, the fillet ash content was significantly (*P* < 0.05) higher in the WBHPA-100 group compared to the WUHPA and CSA groups. Furthermore, the fillet gross energy content in the WBHPA-100 group was significant (*P* < 0.05) higher than in the WUHPA group, but significantly (*P* < 0.05) lower than in the CSA group.

### 3.2. Fillet and Serum Astaxanthin Content

**Figure 1:**
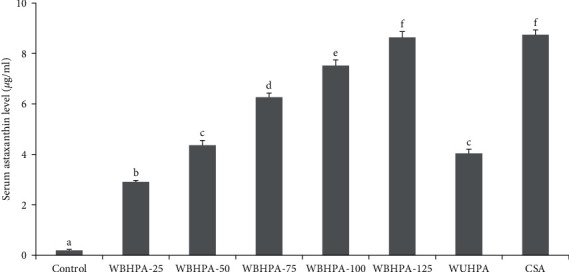
Serum astaxanthin content of rainbow trout *O. mykiss* fed with astaxanthin from different source (means ± SE). Bars with different superscript letters differ significantly (*P* < 0.05).

Increasing dietary WBHPA inclusion level led to a linear increase in serum astaxanthin level (*P* < 0.05; Figure 1). Fillet astaxanthin deposition initially increased and then decreased as the dietary WBHPA inclusion level increased (Table 1), with the highest levels observed in the WBHPA-100 group.

At the 100 mg/kg astaxanthin inclusion level, the serum astaxanthin level in the WBHPA-100 group was significantly (*P* < 0.05) higher than that in the WUHPA group, but significantly (*P* < 0.05) lower than that in the CSA group. A similar trend was observed for fillet astaxanthin content, although no significant difference (*P* > 0.05) was noted between the WBHPA-100 and CSA groups.

### 3.3. Fillet Color

**Figure 2:**
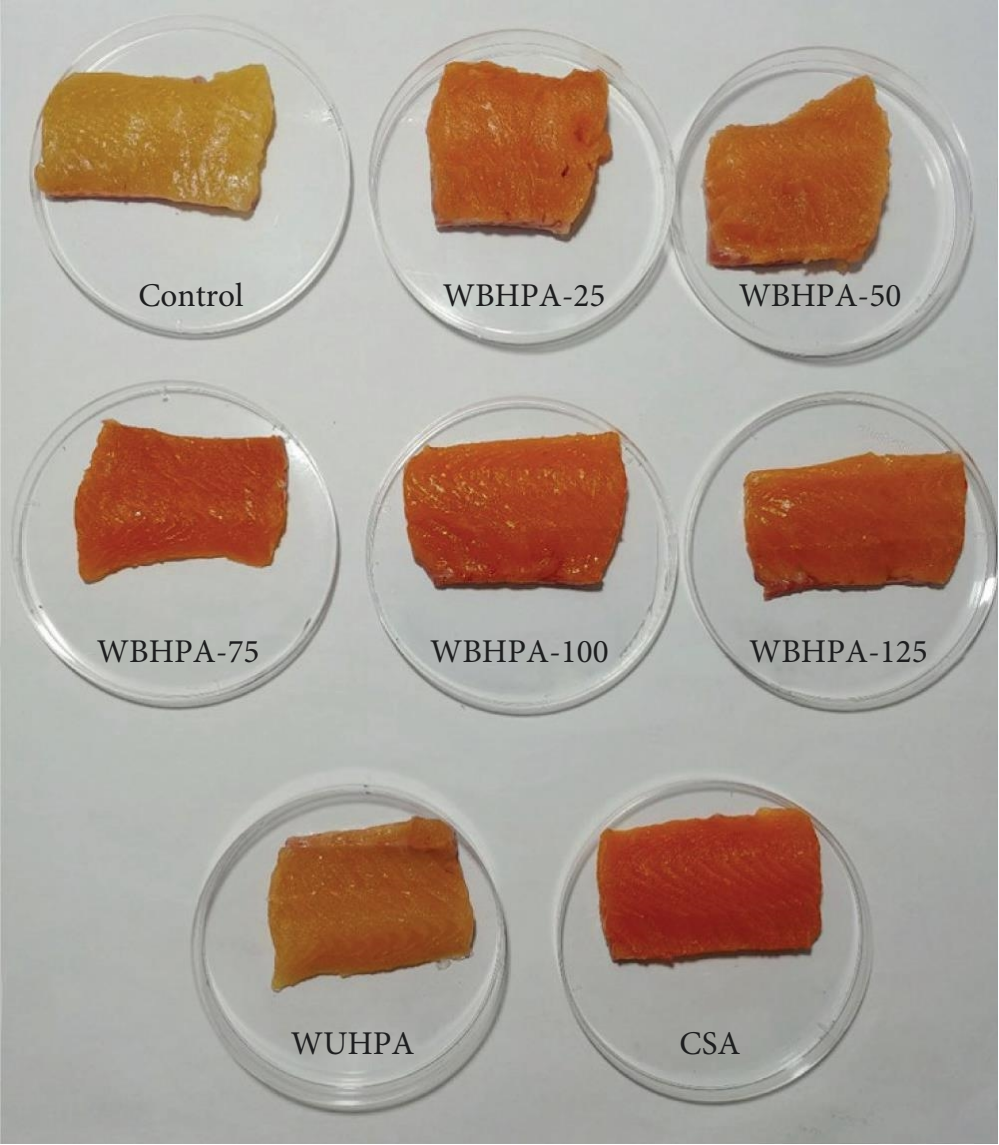
Fillet color of rainbow trout *O. mykiss* fed with astaxanthin from different sources.

Dietary supplementation of 25–125 mg/kg WBHPA significantly (*P* < 0.05) decreased the *L* ^*∗*^ value of dorsal fillet, but significantly (*P* < 0.05) increased the *a* ^*∗*^ value and color card score of dorsal fillets (Table 2 and Figure 2). The *b* ^*∗*^ value of dorsal fillet was significantly (*P* < 0.05) higher in the WBHPA-75, WBHPA-100, and WBHPA-125 groups compared to the control and WBHPA-25 groups. Astaxanthin inclusion from WBHPA significantly (*P* < 0.05) enhanced the *a* ^*∗*^ value of both the lateral line and abdominal region. The lateral line *b* ^*∗*^ value was significantly (*P* < 0.05) lower in the WBHPA-75, WBHPA-100, and WBHPA-125 groups compared to the control group.

The *L* ^*∗*^ value of dorsal fillet in the WBHPA-100 and WUHPA groups was significantly (*P* < 0.05) higher than in the CSA group. The CSA group had the highest values of dorsal fillet *a* ^*∗*^, *b* ^*∗*^, and color card score, while the lowest values were observed in the WUHPA group. The *a* ^*∗*^ value of both the lateral line and abdominal region was higher in the WBHPA-100 group compared to the WUHPA and CSA groups, whereas the *b* ^*∗*^ value of lateral line was lower.

### 3.4. Fillet Texture

With the increase of dietary WBHPA inclusion, the fillet hardness and adhesiveness firstly decreased and then increased, getting the lowest value in the WBHPA-50 and WBHPA-100 groups, respectively (Table 3). Fillet gumminess and tenderness firstly increased and then decreased, and the highest value was found in the WBHPA-100 group. Dietary 50–100 mg/kg WBHPA inclusion significantly (*P* < 0.05) increased the fillet springiness, but decreased the pH value. The fillet chewiness in the WBHPA-75 group was also significantly (*P* < 0.05) higher than that in the control group.

At the 100 mg/kg astaxanthin inclusion level, the fillet adhesiveness and water-holding capacity in the WBHPA-100 group were lower than those in the WUHPA and CSA groups. However, the fillet tenderness in the WBHPA-100 group was significantly (*P* < 0.05) higher than that in the WUHPA and CSA groups.

### 3.5. Fillet Amino Acid Profiles

Dietary WBHPA inclusion generally decreased the concentration of fillet threonine, serine, glycine, and alanine (Table 4). Therein, the concentrations of fillet threonine and serine in the WBHPA-100 and WBHPA-125 groups were significantly (*P* < 0.05) lower than those in the control group. Moreover, the concentrations of fillet glycine and alanine in the WBHPA-100 group was significantly (*P* < 0.05) lower than those in the control group. Moreover, dietary 100 mg/kg astaxanthin inclusion regardless of source significantly (*P* < 0.05) decreased the concentrations of fillet threonine and serine.

### 3.6. Fillet Fatty Acid Profiles

No significant (*P* > 0.05) difference in the fillet fatty acid profiles were observed among the dietary groups (Table 5). Dietary WBHPA inclusion generally increased the fillet 20 : 5n-3 and 22 : 6n-3 contents, but no significant differences were observed (*P* > 0.05). At the 100 mg/kg astaxanthin inclusion level, the highest values of fillet 18 : 0, 18 : 1n-9, 20 : 4n-6, 20 : 5n-3, and 22 : 6n-3 contents were observed in the WBHPA-100 group, while the lowest values were observed in the CSA group.

## 4. Discussion

The color of rainbow trout fillet is a crucial factor in attracting consumer's attention [18]. In this study, rainbow trout fed with CSA exhibited the best fillet pigmentation performance, followed by the WBHPA group, with the WUHPA group showing the least pigmentation. Similarly, another study on rainbow trout found that fish fed with synthetic astaxanthin had higher fillet redness and yellowness compared to those fed with astaxanthin from WBHPA [13]. However, there were no significant differences in the dorsal fillet yellowness between the CSA and WBHPA-100 groups. Moreover, the fillet astaxanthin content in the WBHPA-100 and CSA groups did not differ significantly but was almost five times higher than that in the WUHPA group. These results suggest that the wall-breaking treatment of *H. pluvialis* enhanced the deposition of astaxanthin in the fillet. It was observed that free-state astaxanthin, rather than esterified astaxanthin, was more readily retained in rainbow trout fillet. On the other hand, in red sea bream (*Pagrus major*), esterified astaxanthin was more effective for skin deposition and coloration than free-state astaxanthin [19, 20]. Therefore, the fillet color parameters varied based on the source of astaxanthin and the species of fish, possibly due to species-specific protein and astaxanthin binding [21]. The present study results indicated that dietary inclusion of WBHPA was as effective as CSA in enhancing fillet pigmentation. Additionally, the supplementation of WBHPA in the diet influenced fish color in a dose-dependent manner, particularly by decreasing lightness and increasing redness, which aligns with findings from another study on rainbow trout [14]. Notably, the inclusion of 50–100 mg/kg astaxanthin from WBHPA had the most significant impact on fillet coloration.

Besides visual appeal, fillet quality is also a crucial factor for consumers. Previous researches have demonstrated that fillet quality is significantly influenced by the feed composition [22, 23], which was also supported by the current study. Fillet texture is an important indicator of fillet quality. In a certain range, fillet hardness has been shown to have a positive correlation with fillet fiber density [24, 25] and a negative correlation with fillet fiber diameter [26, 27]. Some researchers argue that higher fillet hardness signifies better fillet quality [28, 29]. However, the inclusion of dietary astaxanthin from both WBHPA (25–100 mg/kg) and CSA was found to decrease the fillet hardness in rainbow trout, suggesting the need for further investigation. Rainbow trout fed with WBHPA exhibited the highest tenderness value and the lowest adhesiveness value in fillet compared to the WUHPA and CSA groups, indicating an enhancement in fillet quality. Fillet tenderness is influenced by factors such as fiber density, fiber diameter, and lipid content. Gumminess is typically associated with hardness and cohesiveness, while chewiness is linked to springiness, cohesiveness, and hardness [30, 31]. To improve the fillet texture, it is recommended to include 75–125 mg/kg astaxanthin from WBHPA in the diet of rainbow trout, as it results in higher value of fillet springiness, gumminess, chewiness, and tenderness, while reducing fillet adhesiveness.

The nutritional value of fillet is another important factor in assessing fillet quality, with the protein and amino acids profile also impacting the nutritional value, flavor, and function of fish fillet [32]. In this study, dietary astaxanthin did not significantly affect the crude protein and crude lipid contents of rainbow trout fillet, regardless of the source and dose of astaxanthin. Similar results were observed in previous studies on rainbow trout fed astaxanthin-supplemented feed [4, 33]. Additionally, dietary inclusion of 75 or 150 mg/kg CSA in a high-fat (18% lipid) diet had limited effects on the fillet nutrient composition of largemouth bass (*Micropterus salmoides*) [34]. Notably, at 100 mg/kg astaxanthin inclusion, the highest fillet gross energy and astaxanthin deposition were observed in the CSA group, followed by the WBHPA-100 group, which was significantly higher than the WUHPA group. This suggests that wall-breaking treatment of *H. pluvialis* can enhance astaxanthin utilization. Fillet, being rich in mitochondria, serves as the primary site of energy production. Previous research has shown that astaxanthin can enhance mitochondrial activity by directly impacting pathways such as AMPK/Sirtuins/PGC-1*α* pathway [35], potentially explaining the increase in fillet gross energy with dietary astaxanthin inclusion. Furthermore, as dietary WBHPA inclusion increased, both fillet gross energy and astaxanthin deposition also increased, with the highest values were observed in the WBHPA-75 and WBHPA-100 groups, respectively. In another study on rainbow trout, fillet astaxanthin concentration linearly increased with dietary CAS supplementation, showing no significant difference among dietary groups [33].

Amino acids, as the fundamental building blocks of protein, play a crucial role in maintaining normal metabolism. Threonine is an essential amino acid for the growth of many fish species [36]. In this study, dietary inclusion of 100 mg/kg astaxanthin significantly decreased the threonine content in rainbow trout fillet. Conversely, a previous study indicated that the threonine content in the dorsal fillet of rainbow trout increased with the dietary addition of 1% *H. pluvialis* [37]. The amino acids responsible for flavor and taste typically include glutamic acid, aspartic acid, phenylalanine, alanine, and glycine [36, 38]. However, in this study, the addition of 100 mg/kg astaxanthin from WUHPA and CSA did not have a significant impact on these flavor-presenting amino acids. Similarly, there was no significant effect on these amino acids when 1% *H. pluvialis* was included in the diets for rainbow trout [37].

The fatty acid profile of fish fillet is influenced by the diet [39]. The present study found no significant impact of varying dietary sources and levels of astaxanthin on the fatty acid composition of rainbow trout fillet. However, previous research has shown that supplementing with CAS increased the levels of 20 : 5n-3 and 22 : 6n-3 but decreased the level of 18 : 2n-6 in rainbow trout fillet [18]. Similarly, CAS supplementation led to an increased in 18 : 3n-3 and a decreased in 18 : 2n-6 in both fillet and liver of olive flounder [5]. Additionally, red porgy (*P. pagrus*) liver showed increased levels of 20 : 5n-3 and 22 : 6n-3 with CAS supplementation [40]. In contrast, astaxanthin from *H. pluvialis* decreased the levels of 18 : 3n-3, 20 : 5n-3, 22 : 6n-3, and 18 : 2n-6 in the abdominal fillet of rainbow trout [37]. The variations in results across studies may be attributed to factors such as fish species, growth stage, and tissue type, indicating the need for further research.

## 5. Conclusions

The wall-breaking treatment of *H. pluvialis* can enhance astaxanthin utilization, leading to improved fillet color, texture, and gross energy deposition. Supplementation with 50–100 mg/kg astaxanthin from WBHPA enhanced the fillet color, while 75–125 mg/kg astaxanthin from WBHPA improved the fillet texture. Based on the experimental results, WBHPA is recommended as a reliable source of astaxanthin in rainbow trout (mean initial weight of 561 g) feed, with the suggested optimal dose being 75–100 mg/kg of diet.

## Figures and Tables

**Table 1:** Effect of astaxanthin from different sources on the fillet proximate composition of rainbow trout *O. mykiss*.

	Control	WBHPA-25	WBHPA-50	WBHPA-75	WBHPA-100	WBHPA-125	WUHPA	CSA
Dry matter (%)	70.29 ± 0.15	69.88 ± 0.04	70.00 ± 0.13	69.28 ± 0.01	71.19 ± 0.43	70.16 ± 0.34	71.46 ± 0.43	70.20 ± 1.11
Crude protein (%)	19.60 ± 0.76	21.34 ± 0.04	22.12 ± 1.23	22.55 ± 0.43	21.34 ± 3.75	20.71 ± 1.68	23.09 ± 3.20	14.96 ± 3.87
Crude lipid (%)	7.33 ± 0.75	7.32 ± 0.56	5.08 ± 2.20	8.02 ± 0.12	7.91 ± 1.45	7.95 ± 0.04	6.18 ± 0.49	6.40 ± 1.44
Ash (%)	2.19 ± 0.14^c^	1.90 ± 0.03^bc^	1.86 ± 0.02^bc^	1.22 ± 0.07^a^	1.85 ± 0.03^b^	1.32 ± 0.02^a^	1.26 ± 0.04^a^	1.39 ± 0.01^a^
Gross energy (KJ/g)	20.61 ± 0.14^a^	24.67 ± 0.35^b^	26.89 ± 0.15^c^	28.83 ± 0.25^d^	26.72 ± 0.06^c^	26.61 ± 0.31^c^	21.20 ± 0.12^a^	29.28 ± 0.15^d^
Astaxanthin (*μ*g/g)	0.39 ± 0.08^a^	6.75 ± 0.49^bc^	8.31 ± 1.05^cd^	12.24 ± 2.66^def^	15.21 ± 0.41^ef^	11.06 ± 0.47^cde^	2.97 ± 0.62^ab^	17.24 ± 0.18^f^

Values are presented as means ± SE (*n* = 3). Means in the same row with different superscripts are significantly different (*P* < 0.05).

**Table 2:** Effect of astaxanthin from different sources on the fillet color of rainbow trout *O. mykiss*.

	Control	WBHPA-25	WBHPA-50	WBHPA-75	WBHPA-100	WBHPA-125	WUHPA	CSA
Dorsal fillet								
*L* ^*∗*^	46.36 ± 0.50^e^	39.88 ± 0.79^cd^	37.90 ± 0.44^abc^	37.34 ± 0.49^ab^	39.39 ± 0.44^bcd^	40.71 ± 0.49^d^	40.48 ± 0.38^d^	36.69 ± 0.67^a^
*a* ^*∗*^	0.75 ± 0.15^a^	10.02 ± 0.34^c^	11.73 ± 0.34^de^	13.19 ± 0.30^ef^	12.08 ± 0.45^e^	10.47 ± 0.26^cd^	4.68 ± 0.31^b^	14.25 ± 0.53^f^
*b* ^*∗*^	12.59 ± 0.35^ab^	12.56 ± 0.3^ab^	14.06 ± 0.35^bc^	15.01 ± 0.42^c^	15.98 ± 0.75^c^	14.96 ± 0.50^c^	11.17 ± 0.41^a^	16.06 ± 0.63^c^
Color card score	20.61 ± 0.14^a^	24.67 ± 0.35^b^	26.89 ± 0.15^c^	28.83 ± 0.25^d^	26.72 ± 0.06^c^	26.61 ± 0.31^c^	21.20 ± 0.12^a^	29.28 ± 0.15^d^
Lateral line								
*L* ^*∗*^	66.51 ± 2.70	65.12 ± 2.17	62.82 ± 1.99	61.62 ± 2.07	62.15 ± 4.08	61.01 ± 0.90	64.26 ± 1.87	65.46 ± 1.25
*a* ^*∗*^	3.16 ± 0.20^a^	6.22 ± 0.23^d^	4.48 ± 0.22^bc^	4.50 ± 0.45^bc^	4.56 ± 0.42^bc^	5.17 ± 0.42^c^	2.84 ± 0.45^a^	3.50 ± 0.37^ab^
*b* ^*∗*^	6.07 ± 0.57^c^	3.94 ± 0.53^abc^	4.22 ± 0.81^abc^	3.45 ± 0.24^ab^	1.89 ± 0.18^a^	1.93 ± 0.17^a^	5.14 ± 0.99^bc^	3.80 ± 0.81^abc^
Abdominal region								
*L* ^*∗*^	81.68 ± 1.96	82.64 ± 1.34	80.95 ± 1.03	77.16 ± 2.01	78.65 ± 2.41	79.44 ± 1.63	81.64 ± 1.36	82.48 ± 2.01
*a* ^*∗*^	−0.34 ± 0.36^a^	1.25 ± 0.39^b^	1.44 ± 0.64^b^	0.93 ± 0.11^b^	1.66 ± 0.41^b^	1.37 ± 0.42^b^	−0.65 ± 0.23^a^	−0.16 ± 0.22^a^
*b* ^*∗*^	4.54 ± 0.58	4.47 ± 0.97	4.17 ± 0.41	4.45 ± 0.44	4.80 ± 0.59	4.63 ± 0.82	4.57 ± 0.53	5.00 ± 0.60

Values are presented as means ± SE (*n* = 3). Means in the same row with different superscripts are significantly different (*P* < 0.05).

**Table 3:** Effect of astaxanthin from different sources on the fillet quality of rainbow trout *O. mykiss*.

	Control	WBHPA-25	WBHPA-50	WBHPA-75	WBHPA-100	WBHPA-125	WUHPA	CSA
Hardness (kg)	2.19 ± 0.11^bc^	1.88 ± 0.13^ab^	1.61 ± 0.11^a^	1.80 ± 0.07^ab^	1.82 ± 0.18^ab^	2.41 ± 0.15^c^	2.22 ± 0.07^bc^	1.67 ± 0.13^a^
Springiness	0.36 ± 0.01^a^	0.41 ± 0.01^ab^	0.47 ± 0.02^b^	0.42 ± 0.01^b^	0.44 ± 0.02^b^	0.44 ± 0.01^b^	0.44 ± 0.03^b^	0.42 ± 0.02^b^
Cohesiveness	0.21 ± 0.02	0.21 ± 0.01	0.22 ± 0.01	0.22 ± 0.01	0.22 ± 0.01	0.20 ± 0.01	0.21 ± 0.01	0.22 ± 0.01
Adhesiveness	−16.44 ± 0.49^b^	−17.19 ± 0.81^b^	−17.59 ± 1.03^b^	−18.45 ± 0.98^ab^	−22.82 ± 1.31^a^	−20.32 ± 1.26^ab^	−18.36 ± 1.31^ab^	−15.93 ± 1.13^b^
Gumminess (g)	333.46 ± 34.53^a^	345.33 ± 22.31^a^	345.77 ± 21.69^a^	425.37 ± 20.18^ab^	475.80 ± 28.10^b^	450.39 ± 19.89^ab^	378.16 ± 22.68^ab^	414.83 ± 36.05^ab^
Resilience	0.10 ± 0.01	0.11 ± 0.01	0.09 ± 0.00	0.10 ± 0.01	0.11 ± 0.01	0.10 ± 0.00	0.10 ± 0.00	0.11 ± 0.01
Chewiness (g)	151.33 ± 18.72^a^	214.14 ± 17.75^ab^	204.24 ± 26.06^ab^	268.84 ± 20.53^b^	227.89 ± 14.14^ab^	231.36 ± 17.90^ab^	178.45 ± 9.90^ab^	225.05 ± 24.09^ab^
Tenderness (*N*)	2.99 ± 0.15^ab^	2.90 ± 0.14^ab^	3.09 ± 0.24^ab^	3.31 ± 0.11^bc^	3.95 ± 0.23^c^	2.30 ± 0.11^a^	2.78 ± 0.25^a^	2.38 ± 0.13^ab^
pH	6.15 ± 0.05^b^	5.91 ± 0.02^a^	5.93 ± 0.10^a^	5.84 ± 0.06^a^	5.94 ± 0.02^a^	5.99 ± 0.03^ab^	5.93 ± 0.01^a^	5.96 ± 0.02^ab^
Water-holding capacity (%)	25.74 ± 0.49^ab^	26.35 ± 0.79^ab^	26.22 ± 1.38^ab^	24.09 ± 1.50^ab^	22.42 ± 0.56^a^	23.46 ± 0.76^a^	31.36 ± 4.24^b^	29.02 ± 0.95^ab^

Values are presented as means ± SE (*n* = 3). Means in the same row with different superscripts are significantly different (*P* < 0.05).

**Table 4:** Effect of astaxanthin from different sources on the fillet amino acid profile of rainbow trout *O. mykiss* (g/100 g).

	Control	WBHPA-25	WBHPA-50	WBHPA-75	WBHPA-100	WBHPA-125	WUHPA	CSA
Essential amino acid								
Arginine	1.31 ± 0.02	1.23 ± 0.01	1.25 ± 0.01	1.25 ± 0.02	1.21 ± 0.04	1.22 ± 0.04	1.21 ± 0.01	1.21 ± 0.02
Threonine	1.04 ± 0.02^b^	0.98 ± 0.02^ab^	0.99 ± 0.01^ab^	0.97 ± 0.02^ab^	0.94 ± 0.03^a^	0.93 ± 0.02^a^	0.94 ± 0.02^a^	0.94 ± 0.02^a^
Valine	1.14 ± 0.01	1.09 ± 0.02	1.08 ± 0.03	1.13 ± 0.00	1.11 ± 0.04	1.11 ± 0.03	1.16 ± 0.02	1.17 ± 0.02
Methionine	0.69 ± 0.01	0.66 ± 0.01	0.67 ± 0.00	0.66 ± 0.01	0.64 ± 0.02	0.64 ± 0.02	0.64 ± 0.02	0.65 ± 0.01
Isoleucine	0.97 ± 0.01	0.92 ± 0.02	0.92 ± 0.02	0.97 ± 0.01	0.95 ± 0.03	0.96 ± 0.03	0.99 ± 0.02	1.01 ± 0.02
Leucine	1.74 ± 0.03	1.66 ± 0.03	1.68 ± 0.01	1.68 ± 0.02	1.63 ± 0.05	1.61 ± 0.04	1.64 ± 0.02	1.65 ± 0.03
Phenylalanine	1.01 ± 0.02	0.96 ± 0.02	0.96 ± 0.01	0.96 ± 0.01	0.93 ± 0.03	0.93 ± 0.03	0.94 ± 0.01	0.93 ± 0.02
Histidine	0.92 ± 0.03	0.87 ± 0.02	0.88 ± 0.01	0.87 ± 0.02	0.87 ± 0.03	0.83 ± 0.03	0.88 ± 0.01	0.86 ± 0.02
Lysine	2.11 ± 0.04	2.02 ± 0.03	2.02 ± 0.02	2.02 ± 0.03	1.97 ± 0.07	1.97 ± 0.05	1.98 ± 0.02	1.98 ± 0.03
Nonessential amino acid								
Aspartic acid	2.26 ± 0.04	2.14 ± 0.04	2.17 ± 0.02	2.16 ± 0.02	2.09 ± 0.07	2.09 ± 0.05	2.13 ± 0.03	2.12 ± 0.04
Serine	0.87 ± 0.03^b^	0.78 ± 0.01^ab^	0.80 ± 0.01^ab^	0.77 ± 0.04^ab^	0.74 ± 0.03^a^	0.73 ± 0.02^a^	0.72 ± 0.01^a^	0.74 ± 0.01^a^
Glutamic acid	3.35 ± 0.04	3.17 ± 0.06	3.22 ± 0.03	3.19 ± 0.07	3.08 ± 0.09	3.12 ± 0.08	3.15 ± 0.04	3.16 ± 0.07
Glycine	1.00 ± 0.03^b^	0.96 ± 0.02^ab^	0.95 ± 0.01^ab^	0.97 ± 0.01^ab^	0.90 ± 0.02^a^	0.98 ± 0.02^ab^	0.93 ± 0.01^ab^	0.95 ± 0.02^ab^
Alanine	1.30 ± 0.01^b^	1.23 ± 0.02^ab^	1.23 ± 0.01^ab^	1.23 ± 0.01^ab^	1.18 ± 0.03^a^	1.21 ± 0.03^ab^	1.20 ± 0.02^ab^	1.21 ± 0.02^ab^
Tyrosine	0.79 ± 0.01	0.75 ± 0.02	0.77 ± 0.01	0.75 ± 0.01	0.74 ± 0.02	0.72 ± 0.02	0.74 ± 0.01	0.73 ± 0.01
Proline	0.89 ± 0.02	0.84 ± 0.02	0.88 ± 0.01	0.90 ± 0.02	0.85 ± 0.03	0.80 ± 0.02	0.81 ± 0.01	0.81 ± 0.03
∑Amino acids	21.36 ± 0.30	20.25 ± 0.32	20.44 ± 0.17	20.45 ± 0.21	19.82 ± 0.60	19.83 ± 0.50	20.05 ± 0.26	20.08 ± 0.36

Values are presented as means ± SE (*n* = 3). Means in the same row with different superscripts are significantly different (*P* < 0.05).

**Table 5:** Effect of astaxanthin from different sources on the fillet fatty acid profile of rainbow trout *O. mykiss* (mg/g).

	Control	WBHPA-25	WBHPA-50	WBHPA-75	WBHPA-100	WBHPA-125	WUHPA	CSA
14 : 0	3.52 ± 0.61	3.30 ± 0.61	4.47 ± 0.90	3.82 ± 0.63	4.34 ± 1.08	4.61 ± 1.19	3.90 ± 0.54	3.18 ± 0.26
15 : 0	0.69 ± 0.08	0.65 ± 0.08	0.79 ± 0.11	0.71 ± 0.09	0.77 ± 0.13	0.81 ± 0.15	0.70 ± 0.07	0.66 ± 0.05
16 : 0	36.30 ± 7.27	31.10 ± 5.33	44.66 ± 8.98	39.36 ± 7.18	40.79 ± 10.25	46.14 ± 11.46	36.90 ± 5.15	30.35 ± 1.74
17 : 0	1.32 ± 0.22	1.28 ± 0.24	1.67 ± 0.30	1.38 ± 0.23	1.51 ± 0.34	1.65 ± 0.36	1.39 ± 0.16	1.16 ± 0.18
18 : 0	12.00 ± 2.41	10.45 ± 1.92	14.79 ± 3.05	12.36 ± 2.22	13.22 ± 3.08	14.81 ± 3.76	11.77 ± 1.67	10.02 ± 1.58
20 : 0	0.55 ± 0.07	0.53 ± 0.07	0.60 ± 0.07	0.55 ± 0.06	0.58 ± 0.08	0.64 ± 0.11	0.53 ± 0.04	0.49 ± 0.03
22 : 0	0.51 ± 0.05	0.46 ± 0.04	0.53 ± 0.06	0.49 ± 0.05	0.41 ± 0.14	0.53 ± 0.07	0.46 ± 0.04	0.42 ± 0.02
23 : 0	2.62 ± 0.33	2.44 ± 0.19	3.13 ± 0.64	2.56 ± 0.35	2.45 ± 0.39	2.94 ± 0.46	2.55 ± 0.24	2.45 ± 0.23
*Σ*SFA	57.50 ± 11.00	50.22 ± 8.46	70.64 ± 14.09	61.24 ± 10.81	64.07 ± 15.46	72.13 ± 17.51	58.21 ± 7.88	48.74 ± 2.73
16 : 1n-7	5.29 ± 1.25	4.24 ± 0.81	5.96 ± 1.33	5.83 ± 1.35	5.93 ± 1.71	6.56 ± 1.85	5.32 ± 0.95	4.27 ± 0.30
17 : 1n-7	0.68 ± 0.09	0.65 ± 0.09	0.79 ± 0.14	0.71 ± 0.07	0.80 ± 0.16	0.80 ± 0.17	0.71 ± 0.08	0.61 ± 0.04
18 : 1n-9c	52.73 ± 13.06	44.54 ± 10.03	67.13 ± 14.94	55.45 ± 12.57	60.22 ± 17.02	66.84 ± 19.64	53.62 ± 8.83	43.86 ± 3.60
20 : 1	2.32 ± 0.54	2.01 ± 0.42	2.58 ± 0.57	2.41 ± 0.39	2.62 ± 0.63	2.82 ± 0.86	2.33 ± 0.36	1.96 ± 0.25
22 : 1n-9	0.68 ± 0.06	0.66 ± 0.07	0.73 ± 0.08	0.66 ± 0.06	0.54 ± 0.18	0.71 ± 0.12	0.68 ± 0.06	0.60 ± 0.04
24 : 1n-9	0.71 ± 0.06	0.74 ± 0.07	0.97 ± 0.19	0.78 ± 0.09	0.93 ± 0.18	0.89 ± 0.09	0.77 ± 0.06	0.73 ± 0.03
*Σ*MUFA	62.42 ± 15.04	52.85 ± 11.49	78.16 ± 17.22	65.83 ± 14.52	71.03 ± 19.82	78.62 ± 22.71	63.42 ± 10.29	52.03 ± 4.20
18 : 2n-6c	75.89 ± 19.07	65.84 ± 15.20	104.41 ± 21.50	82.88 ± 20.31	86.12 ± 23.73	97.92 ± 28.55	79.86 ± 13.26	64.74 ± 5.28
18 : 3n-6	3.69 ± 0.84	3.25 ± 0.72	5.16 ± 1.16	3.90 ± 0.69	4.08 ± 1.24	4.44 ± 1.12	3.65 ± 0.48	3.34 ± 0.39
18 : 3n-3	8.05 ± 1.97	7.02 ± 1.54	11.38 ± 2.29	9.08 ± 2.20	9.61 ± 2.71	10.82 ± 3.19	8.81 ± 1.41	6.95 ± 0.60
20 : 2	3.91 ± 0.90	3.02 ± 0.52	4.29 ± 0.81	3.81 ± 0.72	3.89 ± 0.92	4.66 ± 1.24	3.65 ± 0.60	3.06 ± 0.23
20 : 3n-6	2.48 ± 0.51	2.18 ± 0.33	3.09 ± 0.65	2.53 ± 0.46	2.45 ± 0.54	3.00 ± 0.66	2.29 ± 0.26	2.13 ± 0.11
20 : 4n-6	4.98 ± 0.75	4.82 ± 0.65	6.49 ± 1.42	5.36 ± 0.60	5.67 ± 1.07	6.09 ± 0.99	5.11 ± 0.51	4.71 ± 0.17
20 : 3n-3	0.49 ± 0.11	0.37 ± 0.05	0.49 ± 0.09	0.42 ± 0.09	0.43 ± 0.15	0.52 ± 0.14	0.45 ± 0.08	0.38 ± 0.05
20 : 5n-3	4.13 ± 0.50	4.43 ± 0.75	5.82 ± 1.11	4.69 ± 0.45	5.96 ± 1.11	5.88 ± 1.21	5.06 ± 0.49	4.33 ± 0.36
22 : 2	0.61 ± 0.07	0.44 ± 0.15	0.63 ± 0.08	0.31 ± 0.18	0.55 ± 0.19	0.50 ± 0.26	0.45 ± 0.15	0.55 ± 0.02
22 : 5n-3c	1.65 ± 0.21	1.77 ± 0.27	2.10 ± 0.35	1.79 ± 0.17	2.01 ± 0.37	1.94 ± 0.33	1.76 ± 0.16	1.62 ± 0.14
22 : 6n-3	16.02 ± 1.23	17.42 ± 1.54	22.10 ± 4.58	19.03 ± 1.64	19.45 ± 2.13	21.91 ± 3.09	18.74 ± 1.62	16.95 ± 0.76
*Σ*PUFA	121.90 ± 26.01	111.56 ± 21.59	165.95 ± 33.35	133.80 ± 27.43	140.22 ± 33.90	157.67 ± 39.83	129.83 ± 18.83	108.79 ± 7.87
n-3 PUFA	30.34 ± 3.95	31.01 ± 4.12	41.89 ± 8.21	35.01 ± 4.52	37.46 ± 6.37	41.05 ± 7.50	34.83 ± 3.72	30.24 ± 1.81
n-6 PUFA	87.04 ± 21.15	76.09 ± 16.82	119.15 ± 24.59	94.68 ± 22.05	98.32 ± 26.53	111.45 ± 31.07	90.90 ± 14.47	74.93 ± 5.93
*Σ*n-3/n-6 PUFA	0.38 ± 0.04	0.45 ± 0.06	0.36 ± 0.02	0.39 ± 0.04	0.45 ± 0.09	0.39 ± 0.04	0.39 ± 0.02	0.40 ± 0.01
*Σ*Total fatty acids	241.82 ± 52.02	213.62 ± 41.50	314.75 ± 64.60	260.87 ± 52.72	275.32 ± 69.16	308.42 ± 79.96	251.46 ± 36.93	209.55 ± 14.84

Values are presented as means ± SE (*n* = 3). Means in the same row with different superscripts are significantly different (*P* < 0.05).

## Data Availability

Raw data supporting the conclusions of this manuscript will be made available by the authors, without undue reservation, to any qualified researcher.

## Supplementary Materials

Supplementary Materials Table S1: Ingredients and proximate composition (% dry matter) of eight experimental diets for rainbow trout *O. mykiss*.
