# Supplementary material for: Effects of Different Astaxanthin Sources on Fillet Coloration and Energy Deposition in Rainbow Trout (Oncorhynchus mykiss)
Source: Aquac Nutr. 2024 Mar 25;2024:1664203. doi: 10.1155/2024/1664203 (PMC10984719; doi:10.1155/2024/1664203)
Supplement: Supplementary Materials — Table S1: Ingredients and proximate composition (% dry matter) of eight experimental diets for rainbow trout O. mykiss. [file 1664203.f1.docx]

**Supplementary material**

**Effects of different astaxanthin sources on fillet coloration and energy deposition in rainbow trout (*Oncorhynchus mykiss*)**

Xiaoxue Meng^1^, Fumei Yang^2^, Lulu Zhu^1^, Lingli Zhan^2^, Toru Numasawa^2^**^,^***, and Junming Deng^1^**^,^***

^1^ College of Fisheries, Guangdong Ocean University, Zhanjiang 524088, China.

^2^ Kunming Biogenic Co., Ltd., Kunming 650220, China.

Correspondence should be addressed to Toru Numasawa; [numasawa@bgenic.com](mailto:numasawa@bgenic.com) and Junming Deng; [djunming@163.com](mailto:djunming@163.com).

**Supplementary Table S1** Ingredients and proximate composition (% dry matter) of eight experimental diets for rainbow trout *Oncorhynchus mykiss.*

| Ingredients | Control | WBHPA-25 | WBHPA-50 | WBHPA-75 | WBHPA-100 | WBHPA-125 | WUHPA | CSA |
| --- | --- | --- | --- | --- | --- | --- | --- | --- |
| Fish meal^1^ | 28.00 | 28.00 | 28.00 | 28.00 | 28.00 | 28.00 | 28.00 | 28.00 |
| Soybean meal^1^ | 28.00 | 28.00 | 28.00 | 28.00 | 28.00 | 28.00 | 28.00 | 28.00 |
| Rapeseed meal^1^ | 12.00 | 12.00 | 12.00 | 12.00 | 12.00 | 12.00 | 12.00 | 12.00 |
| Wheat flour | 2.18 | 2.013 | 1.846 | 1.679 | 1.512 | 1.345 | 1.958 | 2.08 |
| *α*-starch | 12.00 | 12.00 | 12.00 | 12.00 | 12.00 | 12.00 | 12.00 | 12.00 |
| Soybean oil | 8.00 | 8.00 | 8.00 | 8.00 | 8.00 | 8.00 | 8.00 | 8.00 |
| Fish oil | 6.00 | 6.00 | 6.00 | 6.00 | 6.00 | 6.00 | 6.00 | 6.00 |
| Soybean lecithin | 0.50 | 0.50 | 0.50 | 0.50 | 0.50 | 0.50 | 0.50 | 0.50 |
| Astaxanthin from wall-broken *H. pluvialis*^2^ | ‒ | 0.167 | 0.334 | 0.501 | 0.668 | 0.835 | ‒ | ‒ |
| Astaxanthin from wall-unbroken *H. pluvialis*^2^ | ‒ | ‒ | ‒ | ‒ | ‒ | ‒ | 0.222 | ‒ |
| Chemically synthesized astaxanthin^3^ | ‒ | ‒ | ‒ | ‒ | ‒ | ‒ | ‒ | 0.10 |
| Vitamin C | 0.02 | 0.02 | 0.02 | 0.02 | 0.02 | 0.02 | 0.02 | 0.02 |
| Ca(H_2_PO_4_)_2_ | 1.00 | 1.00 | 1.00 | 1.00 | 1.00 | 1.00 | 1.00 | 1.00 |
| Choline chloride (50%) | 0.30 | 0.30 | 0.30 | 0.30 | 0.30 | 0.30 | 0.30 | 0.30 |
| Vitamin mixture^4^ | 1.00 | 1.00 | 1.00 | 1.00 | 1.00 | 1.00 | 1.00 | 1.00 |
| Mineral mixture^5^ | 1.00 | 1.00 | 1.00 | 1.00 | 1.00 | 1.00 | 1.00 | 1.00 |
| *Proximate composition* |  |  |  |  |  |  |  |  |
| Dry matter | 90.32 | 91.26 | 90.43 | 91.31 | 91.19 | 90.96 | 89.30 | 90.97 |
| Crude protein | 39.86 | 39.74 | 39.97 | 39.92 | 40.02 | 39.89 | 39.76 | 40.14 |
| Crude lipid | 17.97 | 17.99 | 18.09 | 18.26 | 18.30 | 18.26 | 18.17 | 18.31 |
| Ash | 10.07 | 9.87 | 9.84 | 9.71 | 10.05 | 9.79 | 9.95 | 9.99 |
| Gross energy (MJ/kg DM) | 21.92 | 21.99 | 22.06 | 21.83 | 22.01 | 21.91 | 21.80 | 21.72 |
| Astaxanthin (mg/kg DM) | ND | 24.56 | 48.60 | 72.15 | 95.19 | 117.72 | 95.77 | 94.27 |

^1^Supplied by Kunming Tianyuan Feed Co., Ltd. (Yunnan, China); fish meal, 72.31% crude protein, 10.00% crude lipid; soybean meal, 52.98% crude protein, 0.85% crude lipid; rapeseed meal, 42.93% crude protein, 1.51% crude lipid.

^2^Supplied by Kunming Biogenic Co., Ltd. (Yunnan, China); the purity of astaxanthin from wall-broken or wall-unbroken green algal *H. pluvialis* is 1.5% or 4.5%, respectively.

^3^Supplied by DSM Co., Ltd. (Shanghai, China); the purity of astaxanthin is 10.0%.

^4^Vitamin premix (g/kg mixture): retinyl acetate (2800000 IU/g), 2; cholecalciferol, 0.03; DL-α-tocopheryl acetate, 30; menadione, 3; thiamine hydrochloride, 8; riboflavin, 11; pyridoxine hydrochloride, 8; vitamin B_12_, 0.02; ascorbic acid, 50; folic acid, 1; biotin, 0.1; niacin, 30; calcium D-pantothenate, 32; inositol, 25.

^5^ Mineral premix (g/kg mixture): MgSO_4_•7H_2_O, 180; KI, 1; FeSO_4_•H_2_O, 260; ZnSO_4_•H_2_O, 180; CuSO_4_•5H_2_O, 25; Na_2_Se_2_O_3_, 0.01; MnSO_4_•H_2_O, 180; CoCl_2_•6H_2_O, 0.75.
